# Insulin Receptor Substrate-1 (IRS-1) and IRS-2 expression levels are associated with prognosis in non-small cell lung cancer (NSCLC)

**DOI:** 10.1371/journal.pone.0220567

**Published:** 2019-08-08

**Authors:** Andrew J. Piper, Jennifer L. Clark, Jose Mercado-Matos, Asia N. Matthew-Onabanjo, Chung-Cheng Hsieh, Ali Akalin, Leslie M. Shaw

**Affiliations:** 1 Department of Molecular, Cell & Cancer Biology, University of Massachusetts Medical School, Worcester, Massachusetts, United States of America; 2 Department of Pathology, University of Massachusetts Medical School, Worcester, Massachusetts, United States of America; H. Lee Moffitt Cancer Center & Research Institute, UNITED STATES

## Abstract

The insulin-like growth factor-1 (IGF-1) signaling pathway has been implicated in non-small cell lung cancer (NSCLC) outcomes and resistance to targeted therapies. However, little is known regarding the molecular mechanisms by which this pathway contributes to the biology of NSCLC. The insulin receptor substrate (IRS) proteins are cytoplasmic adaptor proteins that signal downstream of the IGF-1R and determine the functional outcomes of this signaling pathway. In this study, we assessed the expression patterns of IRS-1 and IRS-2 in NSCLC to identify associations between IRS-1 and IRS-2 expression levels and survival outcomes in the two major histological subtypes of NSCLC, adenocarcinoma (ADC) and squamous cell carcinoma (SCC). High IRS-2 expression was significantly associated with decreased overall survival in adenocarcinoma (ADC) patients, whereas low IRS-1 cytoplasmic expression showed a trend toward association with decreased overall survival in squamous cell carcinoma (SCC) patients. Tumors with low IRS-1 and high IRS-2 expression were found to be associated with poor outcomes in ADC and SCC, indicating a potential role for IRS-2 in the aggressive behavior of NSCLC. Our results suggest distinct contributions of IRS-1 and IRS-2 to the biology of ADC and SCC that impact disease progression.

## Introduction

Non-small cell lung cancer (NSCLC) is the most common type of lung cancer and accounts for 85% of all lung cancer cases [1]. Histologically, NSCLC is divided predominantly into two major subtypes, adenocarcinoma (ADC) and squamous cell carcinoma (SCC), which comprise 50% and 40% of NSCLC cases, respectively [1]. Recent genome sequencing has identified distinct molecular alterations that characterize ADC and SCC [2, 3] and that predict favorable response to therapies in lung cancer patients. Most notably, *EGFR*, *ALK*, and *ROS1* are the most clinically significant mutations in ADC and molecular testing of lung adenocarcinomas is now widely recommended by oncologists [4]. Unfortunately, acquired resistance to targeted therapies occurs in many patients [5], and there is a continuing need to identify resistance mechanisms and to develop alternative and second-line therapies.

One signaling pathway that has been implicated in lung cancer outcomes and resistance to targeted therapies is the insulin-like growth factor-1 (IGF-1) signaling pathway [6]. IGF-1 ligand and IGF-1 receptor (IGF-1R) expression are both elevated in NSCLC, and higher IGF-1R expression is associated with reduced overall survival in SCC patients [7]. NSCLC patients with tumors that overexpress both IGF-1R and EGFR have reduced relapse-free survival (RFS) and overall survival (OS) [8, 9]. With regard to resistance, high IGF-1R expression is a negative predictive factor for response to EGFR tyrosine kinase inhibitors (TKIs) [10–12]. The IGF-1R pathway has also been implicated in response to therapies that target ALK fusion proteins [13]. The role of the IGF-1R in resistance to TKIs underscores the importance of understanding the contribution of this signaling pathway to the biology of NCSLC.

The insulin receptor substrate (IRS) proteins are cytoplasmic adaptor proteins that mediate the functional outcomes of IGF-1R signaling [14]. Although IRS-1 and IRS-2 share significant homology, they regulate distinct functional outcomes in tumor cells [15]. Specifically, IGF-1R-dependent signaling through IRS-1 promotes proliferation, whereas signaling through IRS-2 promotes migration, invasion, and glucose metabolism [14, 16]. Although little is known about the clinical significance of the IRS proteins in human lung cancer, IRS-1 has been implicated in signaling in EML4-ALK rearranged NSCLC [17]. In the current study, we evaluated the expression and intracellular localization patterns of the IRS proteins in NCSLC and determined their associations with clinical outcomes in ADC and SCC. Our study reveals distinct expression patterns for IRS-1 and IRS-2 in ADC and SCC and suggests that the expression levels of IRS-1 and IRS-2 may impact NSCLC biology.

## Materials and methods

### Tumor sections

Formalin-fixed, paraffin-embedded tumor sections were obtained from the Pathology Department archives and tumor bank at the University of Massachusetts Medical School. Approval for the study was obtained from the University of Massachusetts Medical School Institutional Review Board (IRB) and a waiver for consent was issued by this committee. The retrospective study population consisted of patients diagnosed between the years of 2000 and 2014 with NSCLC of any stage. Data on tumor size, tumor grade and node status were available for most patients. Mutational status was available for six ADC tumors: K-RAS (3 tumors), EGFR (2 tumors), EML4-ALK (1 tumor). Follow-up data on adjuvant therapy, recurrence-free survival and overall survival were also available. REMARK criteria were used for this study [18].

### Immunohistochemistry

Tissue sections (5 μM) were deparaffinized and rehydrated, and antigen retrieval was carried out in 0.01M citrate buffer, pH 6.0 and heating in a 770-W microwave oven for 14 minutes for slides to be stained for IRS-1 or 0.001M EDTA, pH 8.0 and heating in a steamer for 35 minutes for slides to be stained with IRS-2. For IRS-1, slides were stained with the Dako Autostainer (Dako, Carpinteria, CA) using EnVision+ (Dako) staining reagents as described previously [19, 20]. For IRS-2, slides were blocked with Avidin/Biotin Blocking Kit (Vector Laboratories) followed by 1X Casein Solution for 1 hr. Sections were stained for 1 hour at room temperature with rabbit monoclonal IRS2 (1:400; #EP976Y, Abcam) followed by incubation for 30 min at room temperature with biotin-conjugated goat anti-rabbit IgG (1:200) and developed using Vectastain ABC kit reagents (Vector Laboratories). All slides were treated with DAB enhancer (#K3468; Dako) and counterstained with hematoxylin.

The IRS-1 antibody used in this study has been characterized in previous studies [19, 20]. To validate IRS-2 antibody specificity, MDA-MB-231 cells (ATCC Cell Biology Collection) were infected with lentiviruses containing small hairpin RNAs (shRNA) targeting GFP or IRS-2 (Open Biosystems, Hunstville, AL). MDA-MB-231 cells extracts were immunoblotted as described previously [20, 21]. Cell pellets were fixed in 10% zinc formalin, embedded in paraffin and stained using the same IHC protocol that was used for staining the tissue sections. Non-specific IgG was used as a negative control.

Sections were evaluated for IRS-1 and IRS-2 staining patterns using the following criteria: 1) Cytoplasmic staining was defined as even, diffuse staining throughout the cytoplasm with no clear demarcation of cell borders; 2) Nuclear staining was defined as positive staining within the nucleus, regardless of cytoplasmic staining; 3) Membrane staining was defined as staining along the cell membrane, with or without cytoplasmic staining. Staining intensity was defined as absent (0), low (1), moderate (2) or high (3) throughout the tumor section. The individuals assessing staining patterns were blinded to all prognostic and follow-up data. All cases were reviewed by an expert pathologist to confirm the original diagnosis, and immunohistochemical staining was analyzed by both a pathologist and at least one additional investigator, with no significant disagreement on intensity or patterns of staining. Stained tumor sections were viewed on a Nikon Eclipse E400 microscope (Nikon, Brighton, MI) and photomicrographs were obtained using a SPOT idea 28.2 5.0 Mp Color camera (SPOT Imaging, Sterling Heights, MI).

### Statistical analysis

The Pearson chi-square test and Fisher’s exact test were used to assess for associations between clinical characteristics and IRS staining intensity or patterns. Overall survival (OS) was measured from the date of first cancer diagnosis to the date of death from any cause and was censored from the date of last follow-up for survivors. OS was estimated by the Kaplan-Meier method and assessed by the use of log-rank test for univariate analysis. We used the Cox proportional-hazard model to assess and control the simultaneous contribution of baseline covariates in multivariable analyses. Variables included in the analysis were age (as a continuous variable), gender, race, tumor size, nodal involvement, tumor grade, adjuvant treatments and TNM staging. A two-sided p-value of <0.05 was considered to indicate statistical significance. Statistical analysis was performed using Stata (version 13.1; StataCorp, College Station, TX).

## Results

### IRS expression in normal lung tissue

IRS-1 and IRS-2 expression in normal lung and non-small cell lung cancer were evaluated by immunohistochemistry (IHC). The IRS-1 antibody used in this study has been characterized in previous studies [19, 20]. Antibody specificity for IRS-2 was validated by staining MDA-MB-231 breast carcinoma cells that expressed an IRS-2 specific shRNA (S1A Fig). Parental MDA-MB-231 cells stained positively for IRS-2 and this staining diminished significantly in the knockdown cells (S1B Fig). Negative staining was observed using a non-specific rabbit IgG (S1C Fig).

IRS expression was assessed in normal lung tissue that was present in the tissue sections (S1D Fig). IRS-1 expression was localized to the cytoplasm and nucleus of normal lung tissue, with cytoplasmic expression strongest in the ciliated respiratory epithelium of the bronchioles and nuclear staining featured most prominently in the alveolar epithelium. (S1E Fig). IRS-2 was expressed diffusely in the normal lung tissues, with localization limited to the cytoplasm (S1F Fig), similar to previous observations in the normal breast [20]. IRS-2 expression was also observed in the smooth muscle and immune cell infiltrates.

### IRS expression patterns in non-small cell lung cancer

Sixty-three cases of ADC and forty-four cases of SCC were evaluated for IRS expression and the intensity of IRS staining was scored on a scale of 0 (none) to 3, as described in Materials and Methods. The clinical characteristics of these tumor datasets are shown in Table 1. Data regarding smoking history was not available for either dataset.

**Table 1:** Clinical characteristics of tumor databases.

	ADC	SCC
	n = 63	n = 44
Age (years), no. (%)		
</ = 55	12 (19.0)	4 (9.1)
>55	51 (81)	40 (90.9)
Median age (years)	67	70.5
Sex, no. (%)		
Female	46 (73.0)	15 (34.1)
Male	17 (27.0)	29 (65.9)
Race, no.		
White	60	41
Black	1	1
Other	2	2
Tumor Size (stage), no. (%)		
1	29 (46.0)	10 (22.7)
2	14 (22.2)	14 (31.8)
3	8 (12.7)	8 (18.2)
4	2 (3.2)	1 (2.3)
Unknown	10 (15.9)	11 (25.0)
Node status (stage), no. (%)		
0	49 (77.8)	30 (68.2)
1	1 (1.6)	3 (6.8)
2	4 (6.3)	2 (4.5)
3	0	0
Unknown	9 (14.3)	9 (20.5)
Grade, no. (%)		
1	16 (25.4)	1 (2.3)
2	27 (42.9)	29 (65.9)
3	14 (22.2)	13 (29.5)
Unknown	6 (9.5)	1 (2.3)
Adjuvant therapy, no. (%)		
None	45 (71.4)	33 (75.0)
Chemotherapy	10 (15.9)	9 (20.5)
Radiation	3 (4.8)	0
Chemotherapy&Radiation	5 (7.9)	2 (4.5)
Median Follow-up RCF (months)	15	16
Median Follow-up OS (months)	31.5	29

**Fig 1:**
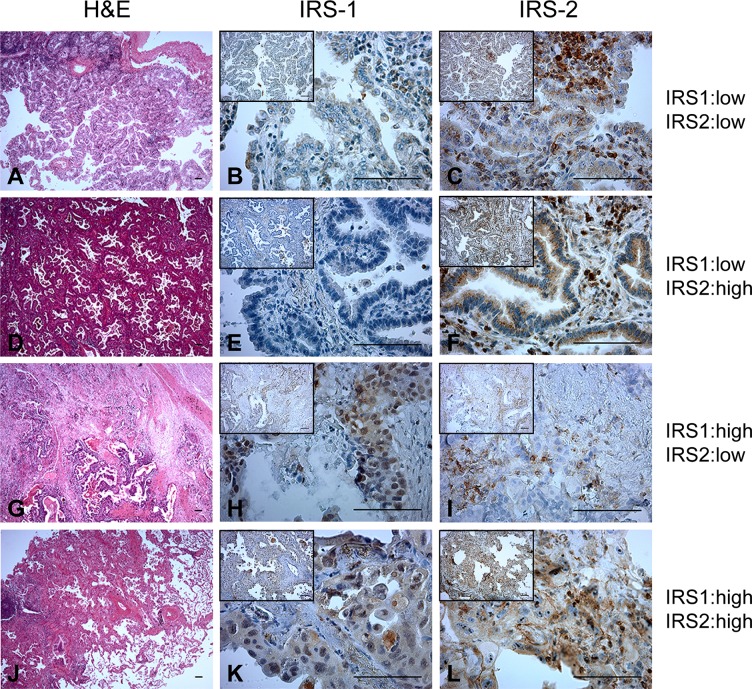
IRS expression in human lung adenocarcinoma. Representative H&E (**A,D,G,J**), IRS-1 (**B,E,H,K**) and IRS-2 (**C,F,I,L**) staining of the same tumors across each row. H&E images, magnification 4X; Larger IRS staining images, magnification 40x; IRS staining inset images, magnification 10X. Scale bar = 50um.

As we and others have reported in breast cancer [19, 20, 22], IRS-1 was expressed in both the cytoplasm and nuclei of ADC. Representative images of IRS staining patterns in ADC are shown in Fig 1, with low expression defined as levels 0 or 1, and high expression defined as levels 2 or 3. IRS-1 expression was solely cytoplasmic in 46% of cases (Fig 1B), both cytoplasmic and nuclear in 23.8% of cases (Fig 1H and 1K) and absent in 30.1% of cases (Fig 1E). We did not observe any ADC tumors with IRS-1 localized only to the nucleus. Low expression of IRS-1 was observed in 83% of ADC cases (Fig 1B and 1E), and high expression was observed in the remaining 17% of tumors (Fig 1H and 1K). Nearly all of the ADC cases (95%) demonstrated cytoplasmic expression of IRS-2. Low cytoplasmic expression of IRS-2 was observed in 52% of tumors (Fig 1C and 1I), and high expression was observed in the remaining 48% of tumors (Fig 1F and 1L). Some degree of IRS-2 membrane staining was also observed in 56% of ADC tumors (Fig 1L). IRS-2 staining was not present in the nuclei of any ADC tumors, similar to our previous observations in invasive breast cancer [20].

**Fig 2:**
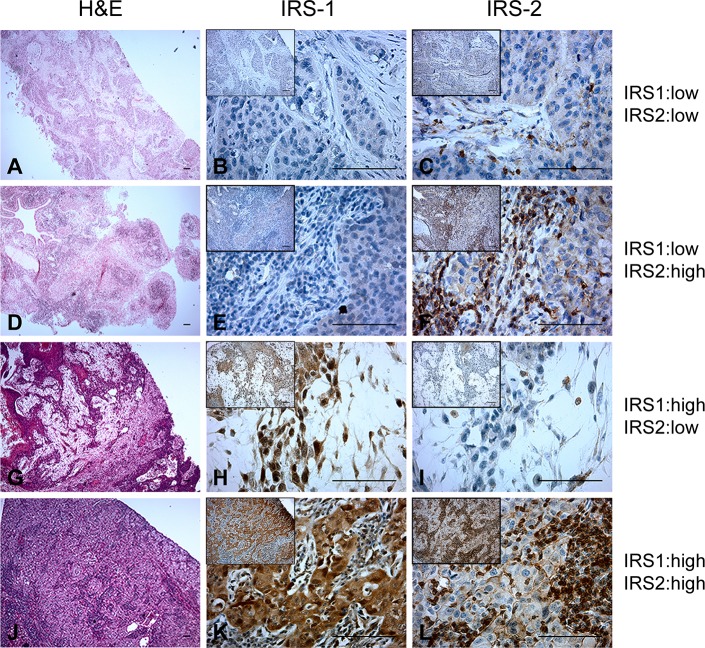
IRS expression in human lung squamous cell carcinoma. Representative H&E (**A,D,G,J**), IRS-1 (**B,E,H,K**) and IRS-2 (**C,F,I,L**) staining of the same tumors across each row. H&E images, magnification 4X; Larger IRS staining images, magnification 40x; IRS staining inset images, magnification 10X. Scale bar = 50um.

Similar to the staining patterns observed in ADC, IRS-1 was expressed in both the cytoplasm and nuclei of SCC. Representative images of IRS staining patterns in SCC are shown in Fig 2, with low expression defined as levels 0 or 1, and high expression defined as levels 2 or 3. In SCC cases, 34.1% of the tumors lacked IRS-1 staining (Fig 2B), 47.7% demonstrated only cytoplasmic staining (Fig 2E), and 15.9% demonstrated both cytoplasmic and nuclear staining (Fig 2H and 2K). Low expression of IRS-1 was observed in 72% of SCC cases (Fig 2B and 2E), and high expression was observed in the remaining 28% of tumors (Fig 2H and 2K). In 91% of SCC cases, IRS-2 expression was present in the cytoplasm. Low expression of IRS-2 was observed in 43% of SCC cases (Fig 2C and 2I), and high expression was observed in the remaining 57% of tumors (Fig 2F and 2L). Some degree of membrane localization of IRS-2 was observed in 73% of tumors (Fig 2F and 2L). Consistent with our findings in ADC, no nuclear staining was observed for IRS-2.

### Correlations with clinical and pathologic characteristics

**Fig 3:**
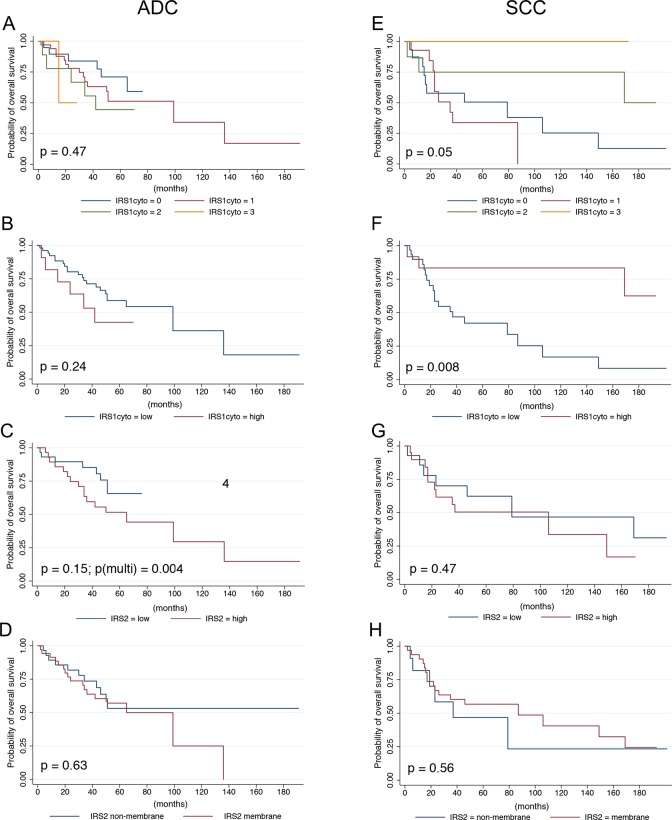
Analysis of IRS staining patterns and overall survival in NSCLC. Kaplan-Meier survival curves showing overall survival (OS) for patients with adenocarcinoma (**A-D**) or squamous carcinoma (**E-H**) as a function of IRS-1 or IRS-2 staining patterns. P-values based on univariate or multivariate (multi) analysis are shown.

A clinical dataset was analyzed to investigate the relationship of IRS staining patterns and intensity to patient characteristics and outcomes (S1 Table). Although a trend toward high cytoplasmic IRS-1 expression and decreased OS was observed, no statistically significant associations with clinical parameters or OS were evident for IRS-1 cytoplasmic or nuclear expression (Fig 3A and 3B). A significant inverse association between IRS-2 cytoplasmic staining intensity and tumor grade was observed when IRS-2 expression was dichotomized to low versus high expression (p = 0.009). Despite this inverse correlation, high IRS-2 expression was associated with a significantly increased risk for death upon multivariate analysis (Table 2, Fig 3C) (HR 35, CI 3.13–389, p = 0.004). IRS-2 membrane expression was not significantly associated with clinical characteristics or overall survival in ADC (Fig 3D).

**Table 2:** Overall survival outcomes analysis for IRS staining.

		Univariate^1^	Multivariate^3^		
	*n*	*P* value	HR^2^	95% CI	*P* value
**ADC**					
IRS-1 staining	*n* = 63				
Negative		0.24	1		
Positive			2.03	0.50–8.32	0.33
IRS-1 cytoplasmic staining	*n* = 63				
High		0.24	1		
Low			0.42	0.12–1.47	0.17
IRS-2 staining	*n* = 58				
Low		0.15	1		
High			34.89	3.13–388.68	0.004
IRS-2 membrane staining	*n* = 63				
Negative		0.63	1		
Positive			1.56	0.53–4.58	0.41
IRS-1:IRS-2	*n* = 58				
Ratio 1		0.61	1		
Ratio 2			49.55	2.11–1161.21	0.02
Ratio 3			1.08	0.04–26.96	0.96
Ratio 4			40.74	2.26–734.09	0.01
**SCC**					
IRS-1 staining	*n* = 43				
Negative		0.25	1		
Positive			0.74	0.16–3.38	0.7
IRS-1 cytoplasmic staining	*n* = 43				
High		0.008	1		
Low			2.23	0.45–10.94	0.32
IRS-2 staining	*n* = 35				
Low		0.47	1		
High			1.4	0.33–5.90	0.64
IRS-2 membrane staining	*n* = 44				
Negative		0.56	1		
Positive			1.17	0.29–4.72	0.83
IRS-1:IRS-2	*n* = 34				
Ratio 1		0.04	1		
Ratio 2			1.25	0.74–189.02	0.77
Ratio 3			0.24	0.03–1.85	0.17
Ratio 4			0	0 - ⚯	

^1^Univariate *P* values were obtained from the log-rank test

^2^Hazard ratios shown were adjusted for age (as a continuous variable) as well as other core clinical variables (as categorical variables) including gender, race, tumor size, nodal involvement, tumor grade, adjuvant treatments and TNM stages.

^3^Multivariate results were obtained from a model that adjusted for the core covariates. The results for IRS staining patterns were obtained from separate models adjusting for the same set of the core covariates.

In SCC, lower IRS-1 expression was significantly associated with worse OS outcomes (Fig 3E; p = 0.05). When dichotomized by low and high cytoplasmic IRS-1 expression, low expression was significantly associated with less nodal involvement (p = 0.009) and tumor grade (p = 0.041) at diagnosis. Despite this negative association with poor prognostic factors, low IRS-1 cytoplasmic staining showed a significant association with worse OS (Table 2, Fig 3F; p = 0.008) on univariate analysis. However, although the effect estimate in the multivariate analysis still pointed in the same direction of the survival difference observed for the univariant analysis (HR 2.23, CI 0.45–10.94, p = 0.32) the findings were no longer significant, likely due to the small sample size. IRS-2 expression was not found to be significantly associated with clinical characteristics, with the exception of gender (p = 0.016), or OS (Table 2, Fig 3G). IRS-2 membrane staining showed a significant association with tumor size at time of diagnosis (p = 0.032), but no significant associations with other clinical characteristics or OS were identified (Table 2, Fig 3H).

**Fig 4:**
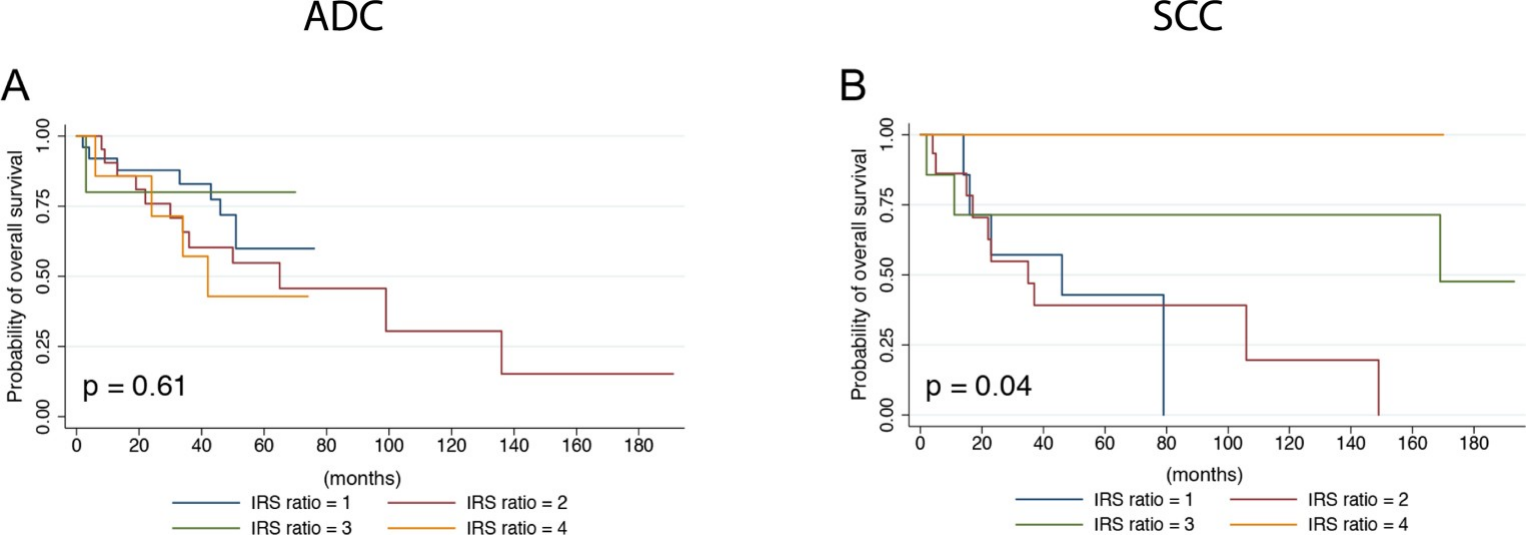
Analysis of combined IRS-1 and IRS-2 expression and overall survival in NSCLC. Kaplan-Meier survival curves showing overall survival (OS) as a function of IRS-1 and IRS-2 expression levels in ADC (**A**) or SCC (**B**). P-values based on univariate analysis are shown.

To examine further how the expression of both IRS-1 and IRS-2 impact outcomes in ADC and SCC, we assessed the association of combined IRS-1 and IRS-2 expression with clinical and pathological characteristics as well as survival outcomes. To do so, tumors were identified as low IRS-1 and low IRS-2 (1), low IRS1 and high IRS-2 (2), high IRS-1 and low IRS-2 (3) and high IRS-1 and high IRS-2 (4). Groups 2 and 4, which both represent high IRS2 expression, were associated with significantly worse OS in ADC after multivariate analysis (Table 2, Fig 4A) (HR 50, CI 2–1161, p = 0.02; HR 41, CI 2.26–734, p = 0.01, respectively). In SCC, Groups 1 and 2, which both represent low IRS-1 expression, were associated with significantly worse OS by univariate analysis (p = 0.04) (Table 2, Fig 4B). However, multivariate analysis did not provide conclusive evidence for survival trends.

## Discussion

In this study, we describe the expression patterns of IRS-1 and IRS-2 in NSCLC and identify associations between IRS-1 and IRS-2 expression levels and survival outcomes in the two major histological subtypes of NSCLC, ADC and SCC. For both ADC and SCC, IRS-1 exhibits diffuse cytoplasmic and combined cytoplasmic and nuclear localization. In contrast, IRS2 is localized diffusely in the cytoplasm or at the cell membrane, but absent from the nucleus. High IRS-2 expression is significantly associated with decreased overall survival in ADC patients, whereas low IRS-1 cytoplasmic expression is significantly associated with decreased overall survival in SCC patients. After adjusting for confounders, tumors with low IRS-1 and high IRS-2 expression were found to be associated with poor outcomes in both ADC and SCC, suggesting that elevated IRS-2 levels may play a role in the aggressive behavior of NSCLC. The hazard ratios reported for these overall survival trends are of high magnitude and carry wide confidence intervals, both suggesting a strong effect size and indicating the need for further validation with a larger cohort to confirm these findings. Taken together, our results support distinct contributions of IRS-1 and IRS-2 to the biology of ADC and SCC that may impact disease progression.

In a previous study of stage I NSCLC, loss of IRS-1 expression was observed more frequently in SCC than other lung cancer types [23], similar to our observation that decreased IRS-1 expression is associated with poor survival outcomes in SCC but not ADC. In that study, decreased IRS-1 expression was more frequently noted in stage 1B tumors when compared with stage IA tumors, but IRS-1 expression was not found to be significantly associated with overall survival [23]. The significant association between low IRS-1 expression and overall survival of SCC patients observed in our current study may reflect the inclusion of later stage tumors in our dataset. Low IRS-1 expression has also been reported to predict poor outcomes specifically for K-RAS mutant ADC [24]. Our analysis, which was neutral for mutational status, showed an opposite survival trend, but not significant association, for IRS-1 expression in ADC. Of note, tumors in our dataset that tested positive for K-RAS mutation (n = 3) all expressed low levels of IRS-1 expression. Future analysis of a larger cohort with greater representation of K-RAS mutations will be necessary to resolve this potential discrepancy in outcomes.

A reduction of IRS-1 expression in more advanced tumors has been observed in other cancer types and supports a suppressive role for IRS-1 signaling in tumor progression. For example, IRS-1 is expressed at high levels in normal breast epithelium and benign breast lesions but expression decreases during the progression to poorly differentiated, invasive carcinomas [19]. In prostate carcinoma cells, IRS-1:IRS-2 ratios are lower in malignant vs. benign prostate tissue and decreased IRS-1 expression is associated with increased motility and invasion, functions associated with later disease stages [25]. In this regard, loss of IRS-1 expression increases mammary tumor metastasis in a mouse MMTV-PyMT tumor model [26]. The recent identification of IRS-1 mutations in NSCLC that suppress migration supports a negative regulatory role for IRS-1 in lung cancer progression [27]. IRS-1 may maintain differentiation and prevent tumor invasion by impeding the epithelial mesenchymal transition (EMT). Specifically, expression of IRS-1 inhibits TGF-ß-induced EMT by a mechanism involving the suppression of the snail and slug EMT transcription factors [28].

High IRS-2 expression was significantly associated with poor OS in ADC, independent of IRS-1 expression levels. Given the ability of IRS-1 to inhibit tumor progression, our finding suggests that alternative mechanisms for suppressing IRS-1 function, rather than expression, may occur in these tumors. IRS-1 signaling can be inhibited through negative feedback pathways that increase IRS-1 serine/threonine phosphorylation, without altering total expression levels [29]. In metastatic mouse mammary tumors, total Irs-1 expression is equivalent to the level of expression observed in non-metastatic tumors, but serine phosphorylation is increased, which inhibits Irs-1 tyrosine phosphorylation and function [26]. Alternatively, IRS-1 and IRS-2 may independently regulate their functional outcomes through the formation of unique scaffolding interactions [30], with the ability of IRS-2 to promote tumor progression being dominant to the suppressive activity of IRS-1. The mechanism for regulating IRS-1 and IRS-2 function may differ between SCC and ADC, with the common outcome that IRS-1 signaling is suppressed and IRS-2 signaling is dominant in both NSCLC subtypes.

The positive association of IRS-2 with poor outcomes in ADC likely reflects its role in regulating tumor cell functions that promote tumor progression. The connection of IRS-2 with tumor progression is substantiated by the observation that IRS-2 protein expression is elevated in later disease stages in many cancers including breast, prostate and peripheral nerve sheath tumors [31–33] and the *IRS2* gene is amplified in additional malignancies including colorectal cancer (CRC) and small cell lung cancer [34, 35]. In mouse models, a role for Irs-2 in progression is demonstrated by the finding that knockout of Irs-2 expression inhibits PyMT-driven mouse mammary tumor metastasis and also suppresses tumor progression in *Pten-/+* mice [21, 31]. Functionally, IRS-2 promotes invasion, an early step in the dissemination of metastatic cells to secondary organs [21]. Our data provide rationale for continued study of the mechanisms by which IRS-1 and IRS-2 contribute to NSCLC and for further validation of the predictive value of the relative levels of IRS-1 and IRS-2 expression for NSCLC outcomes.

## Supporting information

S1 Fig IRS expression in normal human lung tissue. (A) Cell extracts from MDA-MB-231 cells expressing shRNA targeting GFP (shGFP) or IRS-2 (shIRS2) were immunoblotted with the indicated antibodies. (B) MDA-MB-231 cells expressing shRNA targeting GFP (shGFP) or IRS-2 (shIRS2) were fixed in formalin and embedded in paraffin. Sections were stained by IHC using an IRS-2-specific antibody. Magnification 20x. (C) Representative image of IgG staining in NSCLC. (D-F) Representative images of H&E (D), IRS-1 (E) and IRS-2 (F) staining of normal lung tissue. Scale bar = 50um.
(EPS)

S1 Table Clinical data set. Minimal data set used to determine correlations between IRS staining patterns and intensity and patient characteristics and outcomes.
(PDF)

## References

[1] Chen Z, Fillmore CM, Hammerman PS, Kim CF, Wong KK. Non-small-cell lung cancers: a heterogeneous set of diseases. Nat Rev Cancer. 2014;14(8):535–46. Epub 2014/07/25. 10.1038/nrc3775 25056707 PMC5712844

[2] Ding L, Getz G, Wheeler DA, Mardis ER, McLellan MD, Cibulskis K, et al Somatic mutations affect key pathways in lung adenocarcinoma. Nature. 2008;455(7216):1069–75. Epub 2008/10/25. 10.1038/nature07423 18948947 PMC2694412

[3] Cancer Genome Atlas Research N. Comprehensive genomic characterization of squamous cell lung cancers. Nature. 2012;489(7417):519–25. Epub 2012/09/11. 10.1038/nature11404 22960745 PMC3466113

[4] Korpanty GJ, Graham DM, Vincent MD, Leighl NB. Biomarkers That Currently Affect Clinical Practice in Lung Cancer: EGFR, ALK, MET, ROS-1, and KRAS. Front Oncol. 2014;4:204 Epub 2014/08/27. 10.3389/fonc.2014.00204 25157335 PMC4127527

[5] Rotow J, Bivona TG. Understanding and targeting resistance mechanisms in NSCLC. Nat Rev Cancer. 2017;17(11):637–58. Epub 2017/10/27. 10.1038/nrc.2017.84 . 29068003

[6] Pollak M. Insulin and insulin-like growth factor signalling in neoplasia. Nat Rev Cancer. 2008;8(12):915–28. Epub 2008/11/26. 10.1038/nrc2536 . 19029956

[7] Zhao J, Shi X, Wang T, Ying C, He S, Chen Y. The Prognostic and Clinicopathological Significance of IGF-1R in NSCLC: a Meta-Analysis. Cell Physiol Biochem. 2017;43(2):697–704. Epub 2017/09/26. 10.1159/000480655 . 28946136

[8] Ludovini V, Flacco A, Bianconi F, Ragusa M, Vannucci J, Bellezza G, et al Concomitant high gene copy number and protein overexpression of IGF1R and EGFR negatively affect disease-free survival of surgically resected non-small-cell-lung cancer patients. Cancer Chemother Pharmacol. 2013;71(3):671–80. Epub 2013/01/15. 10.1007/s00280-012-2056-y 23314677 PMC3963139

[9] Gately K, Forde L, Cuffe S, Cummins R, Kay EW, Feuerhake F, et al High coexpression of both EGFR and IGF1R correlates with poor patient prognosis in resected non-small-cell lung cancer. Clin Lung Cancer. 2014;15(1):58–66. Epub 2013/11/12. 10.1016/j.cllc.2013.08.005 . 24210543

[10] Suda K, Mizuuchi H, Sato K, Takemoto T, Iwasaki T, Mitsudomi T. The insulin-like growth factor 1 receptor causes acquired resistance to erlotinib in lung cancer cells with the wild-type epidermal growth factor receptor. Int J Cancer. 2014;135(4):1002–6. Epub 2014/01/25. 10.1002/ijc.28737 . 24458568

[11] Murakami A, Takahashi F, Nurwidya F, Kobayashi I, Minakata K, Hashimoto M, et al Hypoxia increases gefitinib-resistant lung cancer stem cells through the activation of insulin-like growth factor 1 receptor. PLoS One. 2014;9(1):e86459 Epub 2014/02/04. 10.1371/journal.pone.0086459 24489728 PMC3904884

[12] Yeo CD, Park KH, Park CK, Lee SH, Kim SJ, Yoon HK, et al Expression of insulin-like growth factor 1 receptor (IGF-1R) predicts poor responses to epidermal growth factor receptor (EGFR) tyrosine kinase inhibitors in non-small cell lung cancer patients harboring activating EGFR mutations. Lung Cancer. 2015;87(3):311–7. Epub 2015/01/27. 10.1016/j.lungcan.2015.01.004 . 25617986

[13] Lovly CM, McDonald NT, Chen H, Ortiz-Cuaran S, Heukamp LC, Yan Y, et al Rationale for co-targeting IGF-1R and ALK in ALK fusion-positive lung cancer. Nat Med. 2014;20(9):1027–34. Epub 2014/09/01. 10.1038/nm.3667 25173427 PMC4159407

[14] Mardilovich K, Pankratz SL, Shaw LM. Expression and function of the insulin receptor substrate proteins in cancer. Cell Commun Signal. 2009;7:14 Epub 2009/06/19. 10.1186/1478-811X-7-14 19534786 PMC2709114

[15] Gibson SL, Ma Z, Shaw LM. Divergent roles for IRS-1 and IRS-2 in breast cancer metastasis. Cell Cycle. 2007;6(6):631–7. Epub 2007/03/16. 10.4161/cc.6.6.3987 . 17361103

[16] Pankratz SL, Tan EY, Fine Y, Mercurio AM, Shaw LM. Insulin receptor substrate-2 regulates aerobic glycolysis in mouse mammary tumor cells via glucose transporter 1. J Biol Chem. 2009;284(4):2031–7. Epub 2008/12/06. 10.1074/jbc.M804776200 19056742 PMC2629099

[17] Kuo AH, Stoica GE, Riegel AT, Wellstein A. Recruitment of insulin receptor substrate-1 and activation of NF-kappaB essential for midkine growth signaling through anaplastic lymphoma kinase. Oncogene. 2007;26(6):859–69. Epub 2006/08/01. 10.1038/sj.onc.1209840 . 16878150

[18] McShane LM, Altman DG, Sauerbrei W, Taube SE, Gion M, Clark GM, et al Reporting recommendations for tumor marker prognostic studies. J Clin Oncol. 2005;23(36):9067–72. Epub 2005/09/21. 10.1200/JCO.2004.01.0454 . 16172462

[19] Schnarr B, Strunz K, Ohsam J, Benner A, Wacker J, Mayer D. Down-regulation of insulin-like growth factor-I receptor and insulin receptor substrate-1 expression in advanced human breast cancer. Int J Cancer. 2000;89(6):506–13. Epub 2000/12/05. 10.1002/1097-0215(20001120)89:6<506::aid-ijc7>3.0.co;2-f . 11102895

[20] Clark JL, Dresser K, Hsieh CC, Sabel M, Kleer CG, Khan A, et al Membrane localization of insulin receptor substrate-2 (IRS-2) is associated with decreased overall survival in breast cancer. Breast Cancer Res Treat. 2011;130(3):759–72. Epub 2011/01/25. 10.1007/s10549-011-1353-1 21258861 PMC3128655

[21] Nagle JA, Ma Z, Byrne MA, White MF, Shaw LM. Involvement of insulin receptor substrate 2 in mammary tumor metastasis. Mol Cell Biol. 2004;24(22):9726–35. Epub 2004/10/29. 10.1128/MCB.24.22.9726-9735.2004 15509777 PMC525494

[22] Sisci D, Morelli C, Garofalo C, Romeo F, Morabito L, Casaburi F, et al Expression of nuclear insulin receptor substrate 1 in breast cancer. J Clin Pathol. 2007;60(6):633–41. Epub 2006/08/03. 10.1136/jcp.2006.039107 16882697 PMC1955087

[23] Han CH, Cho JY, Moon JT, Kim HJ, Kim SK, Shin DH, et al Clinical significance of insulin receptor substrate-I down-regulation in non-small cell lung cancer. Oncol Rep. 2006;16(6):1205–10. Epub 2006/11/08. . 17089038

[24] Metz HE, Kargl J, Busch SE, Kim KH, Kurland BF, Abberbock SR, et al Insulin receptor substrate-1 deficiency drives a proinflammatory phenotype in KRAS mutant lung adenocarcinoma. Proc Natl Acad Sci U S A. 2016;113(31):8795–800. Epub 2016/07/22. 10.1073/pnas.1601989113 27439864 PMC4978299

[25] Heni M, Hennenlotter J, Scharpf M, Lutz SZ, Schwentner C, Todenhofer T, et al Insulin receptor isoforms A and B as well as insulin receptor substrates-1 and -2 are differentially expressed in prostate cancer. PLoS One. 2012;7(12):e50953 Epub 2012/12/20. 10.1371/journal.pone.0050953 23251408 PMC3519512

[26] Ma Z, Gibson SL, Byrne MA, Zhang J, White MF, Shaw LM. Suppression of insulin receptor substrate 1 (IRS-1) promotes mammary tumor metastasis. Mol Cell Biol. 2006;26(24):9338–51. Epub 2006/10/13. 10.1128/MCB.01032-06 17030605 PMC1698550

[27] Gorgisen G, Hapil FZ, Yilmaz O, Cetin Z, Pehlivanoglu S, Ozbudak IH, et al Identification of novel mutations of Insulin Receptor Substrate 1 (IRS1) in tumor samples of non-small cell lung cancer (NSCLC): Implications for aberrant insulin signaling in development of cancer. Genet Mol Biol. 2019;42(1):15–25. Epub 2019/02/27. 10.1590/1678-4685-gmb-2017-0307 30807634 PMC6428125

[28] Shi J, Wang DM, Wang CM, Hu Y, Liu AH, Zhang YL, et al Insulin receptor substrate-1 suppresses transforming growth factor-beta1-mediated epithelial-mesenchymal transition. Cancer Res. 2009;69(18):7180–7. Epub 2009/09/10. 10.1158/0008-5472.CAN-08-4470 . 19738073

[29] Gual P, Le Marchand-Brustel Y, Tanti JF. Positive and negative regulation of insulin signaling through IRS-1 phosphorylation. Biochimie. 2005;87(1):99–109. Epub 2005/03/01. 10.1016/j.biochi.2004.10.019 . 15733744

[30] Fukushima T, Arai T, Ariga-Nedachi M, Okajima H, Ooi Y, Iijima Y, et al Insulin receptor substrates form high-molecular-mass complexes that modulate their availability to insulin/insulin-like growth factor-I receptor tyrosine kinases. Biochem Biophys Res Commun. 2011;404(3):767–73. Epub 2010/12/21. 10.1016/j.bbrc.2010.12.045 . 21168390

[31] Szabolcs M, Keniry M, Simpson L, Reid LJ, Koujak S, Schiff SC, et al Irs2 inactivation suppresses tumor progression in Pten+/- mice. Am J Pathol. 2009;174(1):276–86. Epub 2008/12/20. 10.2353/ajpath.2009.080086 19095950 PMC2631340

[32] Shaw CM, Grobmyer SR, Ucar DA, Cance WG, Reith JD, Hochwald SN. Elevated expression of IRS2 in the progression from neurofibroma to malignant peripheral nerve sheath tumor. Anticancer Res. 2012;32(2):439–43. Epub 2012/01/31. . 22287730

[33] Porter HA, Perry A, Kingsley C, Tran NL, Keegan AD. IRS1 is highly expressed in localized breast tumors and regulates the sensitivity of breast cancer cells to chemotherapy, while IRS2 is highly expressed in invasive breast tumors. Cancer Lett. 2013;338(2):239–48. Epub 2013/04/09. 10.1016/j.canlet.2013.03.030 23562473 PMC3761875

[34] Parsons DW, Wang TL, Samuels Y, Bardelli A, Cummins JM, DeLong L, et al Colorectal cancer: mutations in a signalling pathway. Nature. 2005;436(7052):792 Epub 2005/08/12. 10.1038/436792a . 16094359

[35] George J, Lim JS, Jang SJ, Cun Y, Ozretic L, Kong G, et al Comprehensive genomic profiles of small cell lung cancer. Nature. 2015;524(7563):47–53. Epub 2015/07/15. 10.1038/nature14664 26168399 PMC4861069

