# Supplementary material for: Insulin Receptor Substrate-1 (IRS-1) and IRS-2 expression levels are associated with prognosis in non-small cell lung cancer (NSCLC)
Source: PLoS One. 2019 Aug 8;14(8):e0220567. doi: 10.1371/journal.pone.0220567 (PMC6687170; doi:10.1371/journal.pone.0220567)
Supplement: S1 Table — Minimal data set used to determine correlations between IRS staining patterns and intensity and patient characteristics and outcomes. (PDF) [file pone.0220567.s002.pdf]

Age at Dx	Sex	Race	Tumor Size (1, 2, 3, 4, or X)	Node Stat (0, 1, 2, or X)	Grade (1, 2, 3, or X)	ADJ Tx (Detail) (X= Unk; 0=None; 1= Chemo; 2=Rad; 3=Both)	F-up for RFS (X is unknown; Never canc free = 0)	Ever recurred (Rec =1; No rec = 0; X = Unknown)	F-up for OS	Vital Status (Dead = 0; Alive = 1)	Any IRS2 Membrane	TNM Clinical Stage Group (X = Unk, 1A,1B,2A,2B, 3A,3B)	TNM Path Stage Group (X = Unk/None, 0, 1A,1B,2A,2B,3A,3B)	TNM/CS Mixed Stage Group (X = Unk, 1A,1B,2A,2B,3A,3B)	IRS-1 (0 = none; 1= Cyto; 2= Nuc; 3=both)	IRS-1 Grade Cyto	IRS-1 Grade Nuc	IRS-2 Grade Cyto	IRS-1/IRS-2 ratio (1=low IRS1 and low IRS2, 2=low. IRs1 and high IRS2, 3=high IRS1 and low IRS2, 4=high IRS1 and high IRS2
55	FEMALE	WHITE	2	0	2	1	64	0	75	1	0	1B (1B)	1B	1B	0	0	0	1	1
62	FEMALE	OTHER	1A	0	1	0	X	X	60	1	1	1A (1A)	1B	1B	1	2	0	1	3
49	FEMALE	WHITE	1A	0	1	0	49	0	75	1	1	1A (1A)	1A	1A	0	0	0	3	2
61	FEMALE	WHITE	2	0	3	0	0	1	57	1	1	1B (1B)	3B	3B	1	1	0	3	2
86	MALE	WHITE	X	0	2	0	X	X	65	1	0	99 (X)	1B	1B	0	0	0	2	2
67	FEMALE	WHITE	3	0	3	1	40	0	57	1	1	2B (2B)	3A	3A	0	0	0	1	1
46	FEMALE	WHITE	2	0	1	0	55	0	191	1	0	1B (1B)	X	1B	3	1	1	2	2
71	MALE	WHITE	1	0	1	0	48	0	51	1	1	1 (1)	X	1	0	0	0	3	2
71	MALE	WHITE	X	X	1	0	19	0	136	0	1	99 (X)	1A	1A	1	1	0	2	2
70	FEMALE	WHITE	1A	0	1	0	X	X	52	1	0	1A (1A)	1A	1A	0	0	0	2	2
75	FEMALE	WHITE	1A	0	1	0	33	0	35	1	0	1A (1A)	1A	1A	0	0	0	2	2
62	FEMALE	WHITE	2	2	1	3	8	0	21	1	0	3A (3A)	3A	3A	0	0	0	2	2
54	MALE	BLACK	1B	0	2	0	57	0	46	1	0	1A (1A)	1A	1A	0	0	0	1	1
70	FEMALE	WHITE	1B	0	2	0	35	0	42	0	1	1A (1A)	1A	1A	3	2	3	2	4
75	FEMALE	WHITE	3	0	2	1	21	1	43	0	0	2B (2B)	2B	2B	0	0	0	1	1
67	FEMALE	WHITE	1A	0	2	0	34	0	54	1	1	0	NONE	NONE	3	2	3	2	4
40	FEMALE	ASIAN OTHER/NOS	3	2	X	3	0	1	15	0	1	3A (3A)	3A	3A	3	3	1		
81	FEMALE	WHITE	1B	0	2	0	X	X	51	1	0	0	NONE	NONE	3	2	3	3	4
50	FEMALE	WHITE	1A	0	2	0	12	1	53	1	1	1A (1A)	1A	1A	3	1	2	1	3
66	MALE	WHITE	1	0	2	0	5	0	28	1	1	1 (1)	1B	1B	1	3	0	1	3
63	FEMALE	WHITE	3	0	1	0	12	1	32	1	1	2B (2B)	2B	2B	1	1	0		
69	FEMALE	WHITE	2B	0	1	1	57	0	76	1	1	2A (2A)	2B	2B	0	0	0	1	1
53	FEMALE	WHITE	2A	0	2	0	X	X	73	1	0	2A (2A)	1B	1B	0	0	0		
68	FEMALE	WHITE	1A	0	2	0	13	1	71	1	0	1A (1A)	1A	1A	1	1	0	1	1
80	FEMALE	WHITE	1B	0	2	0	X	X	70	1	0	1A (1A)	1A	1A	1	2	0	1	3
56	FEMALE	WHITE	1B	0	X	0	54	0	74	1	1	1A (1A)	1A	1A	1	1	1	1	1
67	FEMALE	WHITE	1A	0	3	0	56	1	56	1	0	1 (1)	1	1	1	1	0	0	1
57	MALE	WHITE	NONE	NONE	3	3	57	0	74	1	1	99 (X)	X	3A	3	1	2	2	4
62	FEMALE	WHITE	1A	0	1	0	57	0	67	1	1	1 (1)	1	1	3	1	1	1	1
66	MALE	WHITE	1A	0	2	0	45	0	64	1	1	1A (1A)	1B	1B	1	1	0	1	1
66	FEMALE	WHITE	1A	0	X	0	41	0	57	1	1	1A (1A)	1A	1A	0	0	0	2	2
66	FEMALE	WHITE	1A	0	X	0	30	0	52	1	1	1A (1A)	1A	1A	1	1	0	1	1
60	FEMALE	WHITE	2A	2	3	3	33	0	54	1	0	3A (3A)	X	3A	1	1	0	0	1
86	FEMALE	WHITE	1A	0	2	0	33	1	33	1	1	1A (1A)	NONE	1A	1	1	0	1	1
55	FEMALE	WHITE	2A	2	3	0	10	0	13	1	0	3A (3A)	1A	1A	1	1	0	1	1
81	FEMALE	WHITE	2A	0	2	2	4	0	9	1	1	1B (1B)	3A	3A	1	1	0	1	1
67	FEMALE	WHITE	1A	0	2	0	19	0	37	1	0	1A (1A)	1A	1A	0	0	0	1	1
75	MALE	WHITE	2	1	3	0	8	0	30	1	0	2 (2)	2B	2B	1	1	0	1	1
78	FEMALE	WHITE	3	0	3	0	12	1	13	0	0	2B (2B)	2B	2B	1	1	0	1	1
54	FEMALE	WHITE	4	0	2	1	16	1	31	1	0	3A (3A)	1B	1B	1	1	0	1	1
52	FEMALE	WHITE	1A	0	2	0	X	X	22	0	0	1A (1A)	1B	1B	0	0	0		
76	FEMALE	WHITE	3	0	1	0	3	1	22	0	1	2B (2B)	2A	2A	1	1	0	2	2
51	FEMALE	WHITE	2A	0	X	2	X	X	30	0	0	4 (4)	4	4	1	1	0	2	2
79	MALE	WHITE	X	X	1	0	32	0	65	0	1	99 (X)	1	1	0	0	0	3	2
84	MALE	WHITE	X	X	1	0	0	1	50	0	0	99 (X)	1B	1B	3	1	1	2	2
73	FEMALE	WHITE	4	0	2	0	9	1	13	0	1	3B (3B)	1B	1B	3	1	1	2	2
59	MALE	WHITE	2	X	X	1	0	1	34	0	0	1 (1)	X	1	3	2	1	2	4
76	FEMALE	WHITE	3	0	1	0	10	1	24	0	1	2B (2B)	2B	2B	1	2	0	2	4
75	MALE	WHITE	2	0	3	1	X	X	3	0	1	1B (1B)	3A	3A	1	2	0	1	3
66	FEMALE	WHITE	1A	0	2	0	25	1	33	0	1	1A (1A)	1A	1A	3	1	1	1	1
79	MALE	WHITE	X	0	2	0	23	0	34	0	1	99 (X)	1B	1B	3	1	2	2	2
80	FEMALE	WHITE	1A	0	2	0	1	0	2	0	1	1A (1A)	1B	1B	3	1	2	1	1
69	FEMALE	WHITE	NONE	NONE	3	1	14	1	46	0	0	99 (X)	3A	3A	0	Negative	0	1	1
74	FEMALE	WHITE	1A	0	2	1	32	1	51	0	0	1A (1A)	2A	2A	1	1	0	0	1
69	FEMALE	WHITE	NONE	NONE	1	0	99	0	99	0	1	99 (X)	1A	1A	1	1	0	2	2
68	FEMALE	WHITE	1A	0	2	0	4	1	8	0	0	1A (1A)	1A	1A	0	0	0	2	2
68	MALE	WHITE	X	X	3	0	14	1	51	0	1	99 (X)	1A	1A	1	1	0	1	1
67	FEMALE	WHITE	2	0	2	3	3	1	9	0	1	1B (1B)	X	1B	1	1	0	2	2
72	MALE	WHITE	X	X	2	0	36	0	36	0	1	99 (X)	1A	1A	1	1	0	2	2
73	MALE	WHITE	1A	0	3	0	15	1	20	0	1	1A (1A)	3A	3A	1	1	0		
55	MALE	WHITE	3	0	3	0	X	X	19	0	1	2B (2B)	2B	2B	1	1	0	2	2
81	FEMALE	WHITE	1	0	3	2	0	1	4	0	0	1A (1A)	4	4	0	0	0		1
64	FEMALE	WHITE	1A	0	2	1	5	0	6	0	0	1A (1A)	3A	3A	3	2	1	2	4

Adenocarcinoma

Age at Dx	Sex	Race	Tum Size (0, 1, 2, 3, or X)	Node Stat (0, 1, 2, or X)	Grade (1, 2, 3, or X)	ADJ Tx (Detail) (X= Unk; 0=None; 1= Chemo; 2=Rad; 3=Both)	F-up for RFS (X is unknown; Never canc free = 0; M is <1mo Ca Free f/up)	Ever recurred (Rec =1; No rec = 0; X = Unknown)	F-up for OS	Vital Status (Dead = 0; Alive = 1)	Any IRS2 Membrane	TNM Clinical Stage Group (99/X = Unk, 1A,1B,2A,2B,3A,3B)	TNM Path Stage Group (X = Unk, 1A,1B,2A,2B,3A,3B)	TNM/CS Mixed Stage Group (X = Unk, 1A,1B,2A,2B,3A,3B)	IRS-1 (0 = none; 1= Cyto; 2= Nuc; 3=both)	IRS-1 Grade Cyto	IRS-1 Grade Nuc	IRS-2 Grade	IRS-1/IRS-2 ratio (1=low IRS1 and low IRS2, 2=low. IRs1 and high IRS2, 3=high IRS1 and low IRS2, 4=high IRS1 and high IRS2
72	MALE	WHITE	NONE	NONE	3	0	0	1	9	1	0	99 (X)	2B	2B	3	1	2		
62	MALE	WHITE	1A	0	2	0	20	1	48	1	1	1A (1A)	1A	1A	0	0	0		
53	FEMALE	WHITE	3	2	3	0	X	0	65	1	1	3A (3A)	1A	1A	3	2	2	2	4
57	MALE	WHITE	2B	1	3	1	35	0	64	1	1	2B (2B)	3A	3A	1	3	0	1	3
76	MALE	WHITE	3	1	2	0	3	0	66	1	1	3A (3A)	1B	1B	3	3	2	3	4
75	MALE	WHITE	2	0	2	0	77	0	169	0	1	1B (1B)	X	1B	3	2	2	1	3
67	MALE	WHITE	2	0	3	0	88	0	201	1	0	1B (1B)	X	1B	0	0	0		
64	FEMALE	WHITE	1	0	2	0	170	0	172	1	1	1A (1A)	X	1A	3	3	3	1	3
64	MALE	LAOTIAN	3	0	3	0	87	0	193	1	1	2B (2B)	1B	1B	1	2	1	0	3
62	MALE	WHITE	X	X	2	0	129	0	170	1	1	99 (X)	1B	1B	1	2	0	2	4
75	FEMALE	WHITE	X	X	2	0	26	0	149	0	1	99 (X)	1A	1A	0	0	0	2	2
72	MALE	WHITE	3	0	2	0	37	0	58	1	1	2B (2B)	2B	2B	1	1	0	1	1
62	MALE	WHITE	3	0	2	1	3	0	13	1	1	2B (2B)	2B	2B	0	0	0	2	2
74	MALE	WHITE	1B	0	2	0	31	1	42	1	0	1A (1A)	1A	1A	1	1	0	2	2
64	MALE	WHITE	NONE	NONE	2	0	23	1	46	1	0	(NONE)	NONE	NONE	0	0	0	0	1
63	FEMALE	WHITE	X	0	2	1	7	1	37	0	0	99 (X)	3A	3A	1	1	0	2	2
72	MALE	WHITE	2A	0	1	0	X	0	1	1	0	1B (1B)	1B	1B	1	1	0	1	1
77	MALE	WHITE	1A	0	2	0	3	0	4	1	1	1A (1A)	1A	1A	1	1	0	2	2
69	FEMALE	WHITE	3	0	3	0	16	1	22	0	1	2B (2B)	2B	2B	1	1	0	2	2
49	FEMALE	WHITE	X	X	3	1	47	0	72	1	1	99 (X)	2B	3	0	0	0	2	2
59	FEMALE	WHITE	2	0	3	1	0	1	70	1	1	2 (2)	2B	2B	1	2	0	2	4
75	FEMALE	WHITE	1	0	2	0	57	0	87	0	1	1 (1)	1	1	1	1	0		
63	MALE	UNKNOWN	2A	0	2	0	X	0	11	1	0	1B (1B)	1B	1B	1	1	0	1	1
56	FEMALE	WHITE	1	1	3	1	9	1	65	1	1	2A (2A)	2A	2A	3	3	2		
70	MALE	WHITE	2A	0	3	1	49	0	74	1	1	2A (2A)	2A	2A	3	2	1	1	3
54	MALE	WHITE	2A	0	2	1	16	1	23	0	0	1B (1B)	2A	2A	1	1	0	1	1
68	FEMALE	BLACK	3	0	2	1	19	0	37	1	1	2B (2B)	3A	3A	1	1	0	2	2
73	FEMALE	WHITE	2	0	2	0	16	0	79	0	0	1B (1B)	1B	1B	0	0	0	0	1
77	MALE	WHITE	2	0	3	0	9	1	14	0	1	1B (1B)	1B	1B	0	0	0	1	1
71	MALE	WHITE	1A	0	2	3	11	1	19	0	0	1A (1A)	2A	2A	1	1	0		
55	MALE	WHITE	2A	0	2	0	M	0	5	0	1	1B (1B)	1B	1B	1	1	0	2	2
73	FEMALE	WHITE	1	0	2	0	1	0	35	0	1	1A (1A)	1B	1B	1	1	0	2	2
80	MALE	WHITE	1B	0	3	0	M	0	11	0	1	1A (1A)	1B	1B	1	2	1	1	3
72	MALE	WHITE	X	X	3	0	16	0	16	0	1	99 (X)	1B	1B	0	0	0		1
72	MALE	WHITE	X	X	2	0	36	1	46	0	1	99 (X)	1B	1B	0	0	0	1	1
69	MALE	WHITE	NONE	NONE	2	0	19	0	106	0	1	99 (X)	1	1	0	0	0	3	2
73	FEMALE	WHITE	X	X	2	0	6	1	17	0	1	99 (X)	1B	1B	0	0	0	2	2
69	MALE	WHITE	2	2	X	3	M	0	6	0	0	3A (3A)	NONE	3A	0	0	0		
76	MALE	WHITE	4	0	2	0	5	0	17	0	1	3A (3A)	3A	3A			0	2	
77	MALE	WHITE	2A	0	2	0	X	0	4	0	0	1B (1B)	1B	1B	0	0	0	3	2
67	FEMALE	WHITE	3	0	2	0	X	0	15	0	1	2B (2B)	3A	3A	0	0	0	3	2
81	FEMALE	WHITE	2A	0	2	0	M	0	23	0	1	1B (1B)	1A	1A	1	1	0	3	2
80	MALE	WHITE	X	0	2	0	X	0	2	0	1	99 (X)	1A	1A	1	2	0	1	3
78	MALE	WHITE	1A	0	2	0	0	1	26	0	1	1A (1A)	1A	1A	1	1	0		

Squamous Cell Carcinoma
